# Supplementary material for: Spectral analysis of climate dynamics with operator-theoretic approaches
Source: Nat Commun. 2021 Nov 12;12:6570. doi: 10.1038/s41467-021-26357-x (PMC8589855; doi:10.1038/s41467-021-26357-x)
Supplement: Supplementary file 1 — Supplementary information [file 41467_2021_26357_MOESM1_ESM.pdf]

Spectral analysis of climate dynamics with operator-theoretic approaches

Gary Froyland[1], Dimitrios Giannakis[2,3,*], Benjamin R. Lintner[3], Maxwell Pike[3], and Joanna Slawinska[4,5]

[1]School of Mathematics and Statistics, University of New South Wales, Sydney, NSW 2052, Australia
[2]Department of Mathematics and Center for Atmosphere Ocean Science, Courant Institute of Mathematical Sciences, New York University, New York, NY 10012, USA
[3]Department of Mathematics, Dartmouth College, Hanover, NH 03755, USA
[4]Department of Environmental Sciences, Rutgers, The State University of New Jersey, New Brunswick, NJ 08901, USA
[5]Center for Climate Physics, Institute for Basic Science (IBS), Pusan National University, Busan, South Korea
[6]Finnish Center for Artificial Intelligence, Department of Computer Science, University of Helsinki, 00560 Helsinki, Finland
[*]dimitrios.giannakis@dartmouth.edu

SUPPLEMENTARY MATERIAL

Supplementary Note 1

Treatment of the CCSM4 model with periodic seasonal forcing. The CCSM4 model uses fixed (pre-industrial) concentrations of greenhouse gases and perfectly periodic radiative forcing representing the seasonal cycle. In view of this periodic forcing, we explain why our techniques, designed for data generated by autonomous dynamics $\Phi^t : \Omega \to \Omega$, may be formally applied to the CCSM4 model.

Define an augmented state space $S^1 \times \Omega$, where S^1 is a unit radius circle, and let $\theta \in S^1$ denote the phase of the seasonal cycle. The corresponding augmented dynamics $\Psi^t : S^1 \times \Omega \to S^1 \times \Omega$ is given by $\Psi^t(\theta, \omega) = (\theta + 2\pi t/12, \Phi^t_\theta(\omega))$, where the addition in the first component is taken modulo 2π and t is in units of months, and $\Phi^t_\theta : \Omega \to \Omega$ represents (nonautonomous) evolution for duration t initialized in seasonal phase θ. The augmented dynamics Ψ^t is autonomous on $S^1 \times \Omega$ because the periodic seasonal variation, which was previously a forcing for Φ_θ, is now an internal variable for Ψ. The SST images output by the CCSM4 model are in fact functions of the augmented variable (θ, ω) because the sea-surface temperature depends on the seasonal phase θ. In summary, by applying our technique directly to the SST images generated by the CCSM4 model, we are automatically performing the above augmentation to analyze the autonomous dynamics Ψ. Besides the seasonal cycle, other types of periodic forcings of the climate system e.g., the diurnal cycle and tides, are similarly automatically incorporated as internal variables to Ψ^t.

Supplementary Table 1. Dataset attributes and numerical parameter values for the ERSSTv4 and CCSM4 analyses.

	ERSSTv4	CCSM4
Dataset Attributes		
Analysis date range	Jan 1970 – Feb 2020	Jan 0001 – Dec 1300
Climatology date range[a]	Jan 1981 – Dec 2010	Jan 0001 – Dec 1300
Sampling interval Δt	1 month	1 month
Number of snapshots N	602	15,600
SST analysis domain	28°E–70°W, 60°S–20°N	28°E–70°W, 60°S–20°N
Nominal resolution	2°	1°
Number of gridpoints d	4868	44,771
Generator approximation		
Number of delays Q	48	48
Timesteps between delays ℓ	1	1
Kernel bandwidth parameter γ	33	76
Cone kernel parameter ζ	0.995	0.995
Number of kernel eigenfunctions L	400	400
Generator regularization parameter ε	0.001	0.001
Transfer operator approximation		
Number of delays Q	2	2
Timesteps between delays ℓ	12	12
Kernel bandwidth parameter ε	18	65

[a]Used to compute Niño 3.4 indices and SST and surface wind anomaly fields.

Supplementary Table 2. Dataset attributes and numerical parameter values for the L63 analyses.

Dataset Attributes	
Sampling interval Δt	0.01
Number of snapshots N	16,000
Data space dimension d	3
Generator approximation	
Number of delays Q	800
Timesteps between delays ℓ	1
Kernel bandwidth parameter $\tilde{\gamma}$	3.27
Kernel bandwidth parameter γ	0.05
Number of kernel eigenfunctions L	200
Generator regularization parameter ε	0.0005
Transfer operator approximation	
Number of delays Q	1
Timesteps between delays ℓ	1
Kernel bandwidth parameter ε	0.5

Supplementary Table 3. Eigenfrequencies and eigenperiods corresponding to the leading generator eigenfunctions from ERSSTv4 and CCSM4.

	CCSM4			ERSSTv4		
	Frequency (yr^{-1})	Period (yr)	Type	Frequency (yr^{-1})	Period (yr)	Type
g_0	0.000	∞	constant	0.000	∞	constant
g_1	0.997	1.003	annual	0.990	1.010	annual
g_2	−0.997	−1.003	annual	−0.990	−1.010	annual
g_3	1.929	0.518	semiannual	1.916	0.522	semiannual
g_4	−1.929	−0.518	semiannual	−1.916	−0.522	semiannual
g_5	2.546	0.393	triannual	0.000	∞	trend
g_6	−2.546	−0.393	triannual	0.252	3.962	fundamental ENSO
g_7	0.249	4.013	fundamental ENSO	−0.252	−3.962	fundamental ENSO
g_8	−0.249	−4.013	fundamental ENSO	0.970	1.031	trend combination
g_9	0.747	1.339	ENSO combination	−0.970	−1.031	trend combination
g_{10}	−0.747	−1.339	ENSO combination	0.719	1.390	ENSO combination
g_{11}	1.238	0.808	ENSO combination	−0.719	−1.390	ENSO combination
g_{12}	−1.238	−0.808	ENSO combination	2.466	0.406	triannual
g_{13}	1.706	0.586	ENSO combination	−2.466	−0.406	triannual
g_{14}	−1.706	−0.586	ENSO combination	0.000	∞	decadal
g_{15}	2.116	0.473	ENSO combination	1.213	0.825	ENSO combination
g_{16}	−2.116	−0.473	ENSO combination	−1.213	−0.825	ENSO combination
g_{17}	0.347	2.883	3-year ENSO	0.938	1.066	
g_{18}	−0.347	−2.883	3-year ENSO	−0.938	−1.066	
g_{19}	0.602	1.661		0.342	2.923	3-year ENSO
g_{20}	−0.602	−1.661		−0.342	−2.923	3-year ENSO
g_{21}	1.254	0.798		1.538	0.650	
g_{22}	−1.254	−0.798		−1.538	−0.650	
g_{23}	2.429	0.412		1.643	0.609	
g_{24}	−2.429	−0.412		−1.643	−0.609	

Supplementary Table 4. Eigenfrequencies and eigenperiods corresponding to the ENSO eigenfunction of the generator computed from CCSM4, as a function of the number of embedding delays Q. The lag interval ℓ is equal to 1 month.

Number of delays Q	ENSO eigenfrequency (cycles/yr)	ENSO eigenperiod (yr)
12	0.2512	3.9810
24	0.2502	3.9968
48	0.2507	3.9895
96	0.2535	3.9451
192	0.2543	3.9324

Supplementary Table 5. Relative error in the eigenfrequencies $\nu_j = j\alpha/(2\pi)$ with $j = 1,2,3,4$ of a rotation on the circle (harmonic oscillation) of period $T = 2\pi/\alpha = 1$, as a function of the order of finite-difference (FD) approximation of the generator (see Methods). Similarly to the Indo-Pacific SST experiments in the main text, the sampling interval Δt is $T/12$ (i.e., "1 month"), and we use central FD schemes. The eigenfrequencies ν_j are thus analogous to the annual cycle frequency and its harmonics in Fig. 3 and Supplementary Table 3. In particular, the approximation of ν_3 by a fourth-order scheme (highlighted in bold) is analogous to the triannual frequency. In Fig. 3 and Supplementary Table 3, the triannual frequency has a $\simeq 0.15$ relative error, which is comparable to the error highlighted below. The results in this table demonstrate that the error in the approximate eigenfrequencies can be reduced by increasing the order of the FD approximation at a fixed sampling interval.

	Order of FD approximation			
ν_j	2	4	6	8
1	0.0483	0.0092	0.0102	0.0134
2	0.1758	0.0417	0.0176	0.0151
3	0.3655	**0.1567**	0.0755	0.0428
4	0.5879	0.3839	0.2631	0.1869

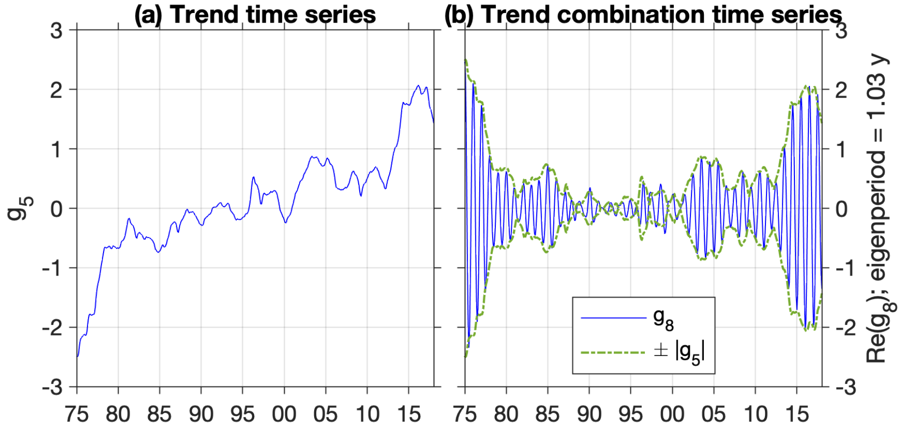

Supplementary Figure 1. Time series associated with eigenfunctions of the generator g_5 (a) and g_8 (b) recovered from ERSSTv4, representing climate change trend and the product (combination) of the annual cycle with the warming trend, respectively. The trend time series in Panel (a) has a manifestly nonstationary behavior, which is qualitatively consistent with a number of large scale features of climate change occurring in the past decades[1]. These features include (i) periods of rapid increase during the mid to late 1970s, early to mid 2000s, and early to mid 2010s; (ii) a more gradual increase from 1980 to 2000; and (iii) a warming "hiatus" during the mid 2000s to mid 2010s. The trend combination time series has the structure of an amplitude-modulated wave with a carrier frequency of approximately 1 cycle per year and a low-frequency modulating envelope with amplitude equal to the modulus of the trend time series.

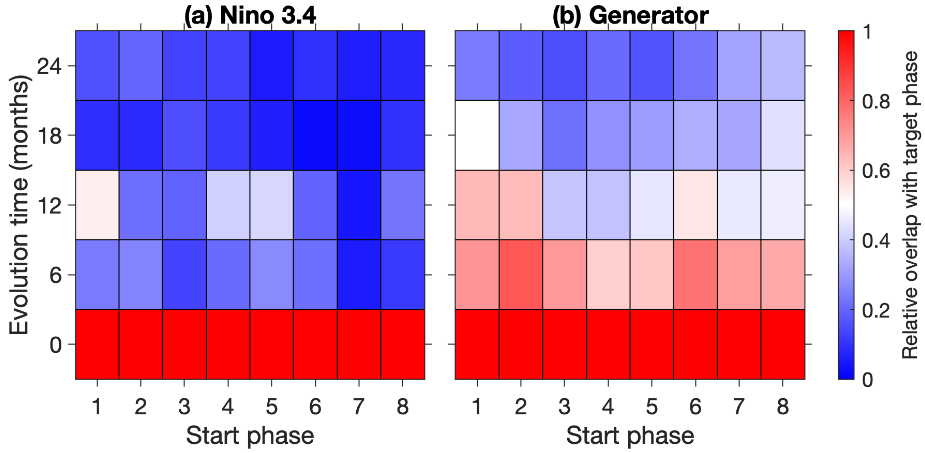

Supplementary Figure 2. Equivariance statistics of the ENSO phase evolution in CCSM4 based on (a) lagged Niño 3.4 indices and (b) the generator. Colors show the number of overlap samples between the image of a phase i_{start} (horizontal axes) under forward evolution by $k \times 6$ months, where $k \in \{0,1,2,3,4\}$, and the target phase $i_{\text{target}} = [(i_{\text{start}} - 1) + (k-1)] \mod 8$ as a fraction of the number of samples, $m = 200$, in each phase.

Supplementary References

1. Lenssen, N. J. L. *et al.* Improvements in the GISTEMP uncertainty model. *J. Geophys. Res. Atmos.* **124**, 6307–6326, DOI: 10.1029/2018JD029522 (2019).
