# Supplementary material for: Spectral analysis of climate dynamics with operator-theoretic approaches
Source: Nat Commun. 2021 Nov 12;12:6570. doi: 10.1038/s41467-021-26357-x (PMC8589855; doi:10.1038/s41467-021-26357-x)
Supplement: Supplementary file 3 — Description of Additional Supplementary Files [file 41467_2021_26357_MOESM3_ESM.pdf]

Description for Additional Supplementary Files

Title: Supplementary Movie 1

Description: Evolution of the leading EOF (covariance) eigenfunctions (a-d) and transfer operator eigenfunctions (e-h), as sampled along a dynamical trajectory of the Lorenz 63 (L63) chaotic system. See the caption of Fig. 2 in the main text for a description of the content of each movie panel.

Title: Supplementary Movie 2

Description: Rectification of a variable-speed oscillator by eigenfunctions of the generator. See the caption of Fig. 4 in the main text for a description of the content of each movie panel.
